# The complete mitochondrial genome of Indian gaur, *Bos gaurus* and its phylogenetic implications

**DOI:** 10.1038/s41598-020-68724-6

**Published:** 2020-07-20

**Authors:** Ranganathan Kamalakkannan, Karippadakam Bhavana, Vandana R. Prabhu, Dhandapani Sureshgopi, Hijam Surachandra Singha, Muniyandi Nagarajan

**Affiliations:** Department of Genomic Science, School of Biological Sciences, Central University of Kerala, Kasaragod, Kerala 671316 India

**Keywords:** Evolutionary genetics, Phylogenetics

## Abstract

The gaur is the largest extant cattle species and distributed across South and Southeast Asia. Around 85% of its current global population resides in India, however there has been a gradual decrease in the gaur population over the last two decades due to various anthropogenic activities. Mitochondrial genome is considered as an important tool for species identification and monitoring the populations of conservation concern and therefore it becomes an obligation to sequence the mitochondrial genome of Indian gaur. We report here for the first time 16,345 bp mitochondrial genome of four Indian gaur sequenced using two different approaches. Mitochondrial genome consisted of 13 protein-coding genes, 2 rRNA genes, 22 tRNA genes, and a control region. Among the 37 genes, 28 were positioned on the H-strand and 9 were positioned on the L-strand. The overall base composition appeared to be 33.5% A, 27.2% T, 25.9% C and 13.4% G, which yielded a higher AT content. The phylogenetic analysis using complete mitochondrial genome sequences unambiguously suggested that gaur is the maternal ancestor of domestic mithun. Moreover, it also clearly distinguished the three sub species of *B. gaurus* i.e. *B. gaurus gaurus, B. gaurus readei* and *B. gaurus hubbacki*. Among the three sub species, *B. gaurus gaurus* was genetically closer to *B. gaurus readei* as compared to *B. gaurus hubbacki*. The findings of our study provide an insight into the genetic structure and evolutionary history of Indian gaur.

## Introduction

The gaur*, Bos gaurus* also known as “Indian bison” is the largest living wild cattle species belonging to the family Bovidae^1^. The historical distribution of gaur ranged throughout much of the mainland South and Southeast Asia. But, currently it occurs in a few Asian countries such as Bangladesh, Bhutan, Cambodia, China, Malaysia, Myanmar, Nepal, Thailand, and Vietnam, with about 85% of its total population surviving in India^2^. Gaur population has declined drastically in almost its entire geographical range primarily due to habitat loss, poaching for horn and meat, diseases and competition for food resources^1,2^. As a result gaur has been categorized as vulnerable species by the IUCN^3^ and protected under schedule I of the wild life (protection) Act 1972 in India. Therefore conservation of gaur populations is pertinent.

The *B. gaurus* has been classified into several subspecies by different researchers elucidating ambiguities in their taxonomy**.** Lydekker^4,5^ reported three subspecies of gaur based on morphological descriptions namely *Bos gaurus gaurus,* which inhabits in India, Nepal and Bhutan, *Bos gaurus readei*, which inhabits in Cambodia, southern China, Lao PDR, Viet Nam, Myanmar, and Thailand and *Bos gaurus hubbacki,* which inhabits in Malaysia. Hubback^6^ opined the possibility for the presence of two types of gaur in Malaysia, one with no dewlap and one possessing well developed dewlap. Recently, Groves^7^ & Groves and Grubb^8^ proposed two sub species; *B. gaurus gaurus* which inhabits in India and Nepal; *B. gaurus laosiensis* which inhabits in Cambodia, Lao PDR, west Malaysia, Myanmar, Thailand, and Vietnam, based on skull and horn size. Another subspecies *B. gaurus sinhaleyus* has been recorded from Sri Lanka which is now extinct^9^. Notably, all the above mentioned studies have classified the subspecies based on morphological/skull features of only a few specimens which confound the taxonomic status of gaur. Considering the phenotypic differences, the International Union for Conservation of Nature (IUCN) has recognized only two sub species of gaur; *B. gaurus gaurus* and *Bos gaurus laosiensis*. However, there is no adequate genetic data to confirm the validity of sub species defined by the morphological data.

It is widely believed that gaur is the ancestral species of domestic mithun (*Bos frontalis*)^10,11^. Nevertheless, a few studies have suggested that mithun is a hybrid descendant of gaur and domestic cattle^12,13^ while a few others have reported mithun as a descendant of an unknown wild bovine which is already extinct^14,15^. Therefore, the origin of domestic mithun remains unresolved. Considering the above facts, genetic characterization of gaur holds great importance. Mitochondrial genome has been widely used to study the evolutionary and phylogenetic relationship of various species due to its high mutation rate and lack of recombination^16–19^. In the present study for the first time, we sequenced the complete mitochondrial genome of four Indian gaur sampled from different places of India. These sequences were analysed with that of Cambodian gaur, Malayan gaur and mithun to resolve the subspecies classification of gaur and shed light on the domestication history of mithun.

## Materials and methods

### Sample collection

In the present study four Indian gaur samples were used. Of the four samples, two were fresh dung samples (sample ID: GR01 & GR02) of free-ranging gaur collected from unprotected forest areas in Karnataka, India. The dung samples were collected within a few minutes of defecation by watching the gaur from a distance. The remaining two were blood and muscle samples of the dead gaur obtained from Arignar Anna Zoological Park, Vandalur, Chennai, (sample ID: GR03), Tamil Nadu and Periyar National Park, Idukki, Kerala, India (sample ID: GR04) respectively. Samples were collected with the help of forest officials/veterinarians for which necessary approval was taken from the concerned state forest departments. The collected dung and tissue samples were preserved in absolute ethanol and stored at − 20 °C until DNA extraction.

### Mitochondrial genome sequencing from dung samples

Genomic DNA from the dung samples (GR01 & GR02) was extracted using QIAamp DNA Stool Mini Kit (Qiagen, Germany) as per the manufacturer’s instructions with few modifications. In brief, 1.5 ml buffer ASL was added to 250 mg dung sample (which contains the mucous layer), vortexed for 1 min, and incubated at 60 °C for 3 h. Subsequently the sample was centrifuged at 14,000 rpm for 3 min and the supernatant was transferred to a new micro centrifuge tube to which one InhibitEX tablet was added and vortexed for 1 min. Following 12 min centrifugation at 14,000 rpm, the supernatant was collected in a new micro centrifuge tube containing 20 µl Proteinase K and 600 µl buffer AL was added which was then vortexed for 15 s and incubated at 70 °C for 15 min. After the incubation, 600 µl absolute ethanol was added to the lysate and mixed thoroughly by vortexing. The lysate was transferred to the QIAamp spin column and centrifuged at 14,000 rpm for 1 min. Followed by, the QIAamp spin column was washed with supplied buffers AW1 and AW2 by centrifugation at 12,000 rpm for 1 min. Then, a high spin (14,000 rpm for 1 min) was given to dry the column membrane. The purified DNA was eluted twice (50 µl per elution) in buffer AE. The quality and quantity of the eluted DNA were checked by agarose gel electrophoresis and NanoDrop One (Thermo Fisher Scientific, USA) respectively.

The complete mitochondrial genome was amplified using 23 sets of overlapping primers^20^ (Supplementary Information Table S1). PCR amplifications were performed in 25 µl reaction mixture which included 80 ng of genomic DNA, 12.5 µl master mix (Promega, USA) and 2 µl (10 pmol) of each primer. The following conditions were applied to the PCR: 5 min initial denaturation at 94 °C followed by 30 cycles of denaturation at 94 °C for 1 min, annealing at 46–59 °C for 1 min, extension at 72 °C for 1 min; and the final extension at 72 °C for 7 min. The amplified PCR products were purified using QIAquick PCR Purification Kit (Qiagen, Germany) as per manufacturer’s instruction and sequenced using both forward and reverse primers. Sequencing was performed in a 10 μl scale using the BigDye Terminator v3.1 Cycle Sequencing Kit (Applied Biosystems, Thermo Fisher Scientific, USA), which included 1 μl ready reaction mix, 1.5 μl sequencing buffer, 10 pmol primer and 200 ng PCR product. The following thermal cycle was applied for the amplification: 96 °C for 1 min, followed by 25 cycles of 96 °C for 10 s, 55 °C for 5 s and 60 °C for 4 min. The excess dye labelled terminators and buffers were removed by EDTA/ethanol precipitation at room temperature. Followed by DNA was denatured by adding 10 μl formamide at 95 ºC for 4 min. Sequencing was performed on ABI 3730XL DNA analyzer (Applied Biosystems, USA). The generated sequencing data was analyzed with Sequencing Analysis Software (Applied Biosystems, USA).

### Mitochondrial genome sequencing from tissue samples

Genomic DNA from tissue samples (blood-GR03 and muscle-GR04) was extracted using DNeasy Blood and Tissue Kit (Qiagen, Germany) as specified by the manufacturer. The complete mitochondrial genome was amplified by long-range PCR using two overlapping sets of primers (5′-AATATGCTCGCCATCATTCC-3′, 5′-ATTGCAGAGGGAAGTCATGG-3′) and (5′-TCACCAGCATAATTCCCACA-3′, 5′-GGCATGTCACCAAGGAGAGT-3′). PCR was performed in 50 μl reaction mixture containing 10 μl of 5XPrimeSTAR GXL Buffer (Takara, Japan), 4 μl of dNTPs (2.5 mM each), 4 μl of each primer (15 pmol), 1 μl of PrimeSTAR GXL DNA Polymerase (Takara, Japan), 23 μl of nuclease free water and 4 μl of DNA template (300 ng). The following conditions were applied for the PCR: 94 °C for 5 min; 30 cycles of 94 °C for 1 min, 68 °C for 1 min and 68 °C for 10 min; 68 °C for 10 min.

The paired end (PE) libraries were prepared using NEBNext Ultra DNA Library Prep Kit (New England BioLabs, USA) as per the manufacturer’s instructions. In brief, the two amplified DNA fragments were pooled together in equimolar concentration and sonicated to a size of 300 bp using Covaris M220 (Covaris, USA). Subsequently the DNA fragments ends were repaired, ‘A’ tailed and ligated to indexed adapters. The resulting 300 bp adaptor-ligated DNA fragments were selected using sample purification beads and enriched through PCR amplification. The purity, size and concentration of the amplified libraries were analysed by Bioanalyzer (Agilent, USA). Finally, the PE libraries (2 × 150 bp) were sequenced on Illumina HiSeq X10 platform (Illumina, USA).

### Mitochondrial genome sequence analysis

A total of 324,110 and 610,358 sequence reads were generated for the samples GR03 and GR04 respectively. The adaptor sequences were removed from the raw reads by Cutadapt v1.8^21^. Further Sickle^22^ and FastUniq^23^ were used to remove low quality and duplicate reads respectively. After quality filtering, high quality reads were assembled using de novo assembly and reference based assembly (de novo assembled GR04 sequence was used as reference for GR03) using SeqManNGen (DNASTAR, USA) assembler. Similarly, the sequences generated through Sanger method was assembled using SeqManPro (DNASTAR, USA). The assembled mitochondrial genome sequences were edited and aligned using the software MegAlign Pro (DNASTAR, USA). MITOS web server was used to annotate the mitochondrial genomes^24^ followed by NCBI ORFfinder (https://www.ncbi.nlm.nih.gov/orffinder) and BLAST (https://blast.ncbi.nlm.nih.gov) were used to validate the annotations. Mitochondrial genome map was constructed using OGDRAW with default parameters^25^. The tRNA secondary structures were analyzed by tRNAscan-SE^26^ and MITOS web servers^24^. Nucleotide composition, Relative Synonymous Codon Usage (RSCU) values and genetic divergence between species were calculated using the software MEGA^27^. The AT and GC skewness were calculated as follows: AT skew = (A – T)/(A + T) and GC skew = (G – C)/(G + C)^28^. The overlapping regions and intergenic spacers between the genes were manually calculated. To ascertain the genetic relationship of Indian gaur with other *Bos* species a Bayesian phylogenetic tree was constructed using the general time reversible model on MrBayes^29^. The MCMC chains were run for 10 × 10^6^ cycles. A total of 20,000 trees were sampled, and a 50% majority rule consensus tree was obtained with burnin = 5,000. The Maximum parsimony (MP) tree was constructed using the software MEGA with 5,000 bootstrap value^27^. The analysis of molecular variance (AMOVA) was calculated with the software ARLEQUIN^30^. The figures were drawn/edited using Inkscape 1.0 (https://inkscape.org).

## Results and discussion

### Mitochondrial genome organization

**Figure 1:**
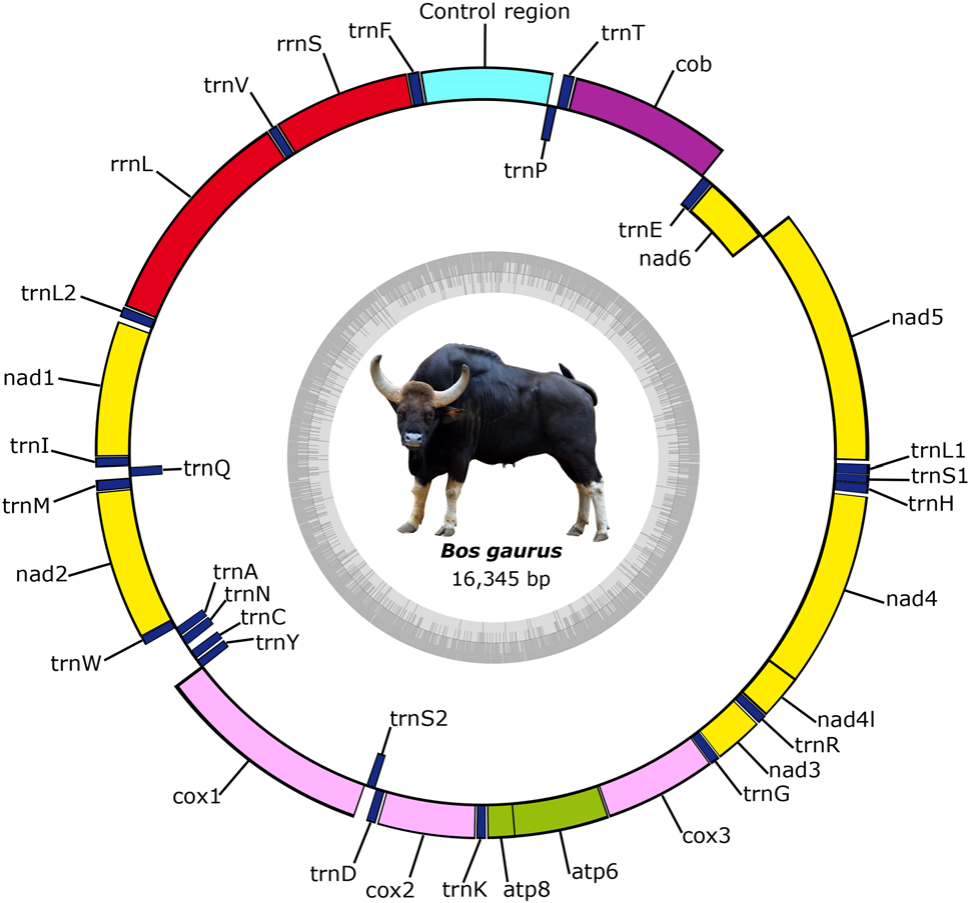
The mitochondrial genome map of Indian gaur. The colored blocks outside the circle denote 28 genes encoded on the H-strand and the colored blocks inside the circle denote the remaining 9 genes encoded on the L-strand. The total GC content of the mitochondrial genome is represented by an inner ring. The mitochondrial genome map was generated using the web server OGDRAW^25^. The Indian gaur photograph was taken by the fourth author Dhandapani Sureshgopi. The final figure was edited manually in Inkscape 1.0. (https://inkscape.org).

**Figure 2:**
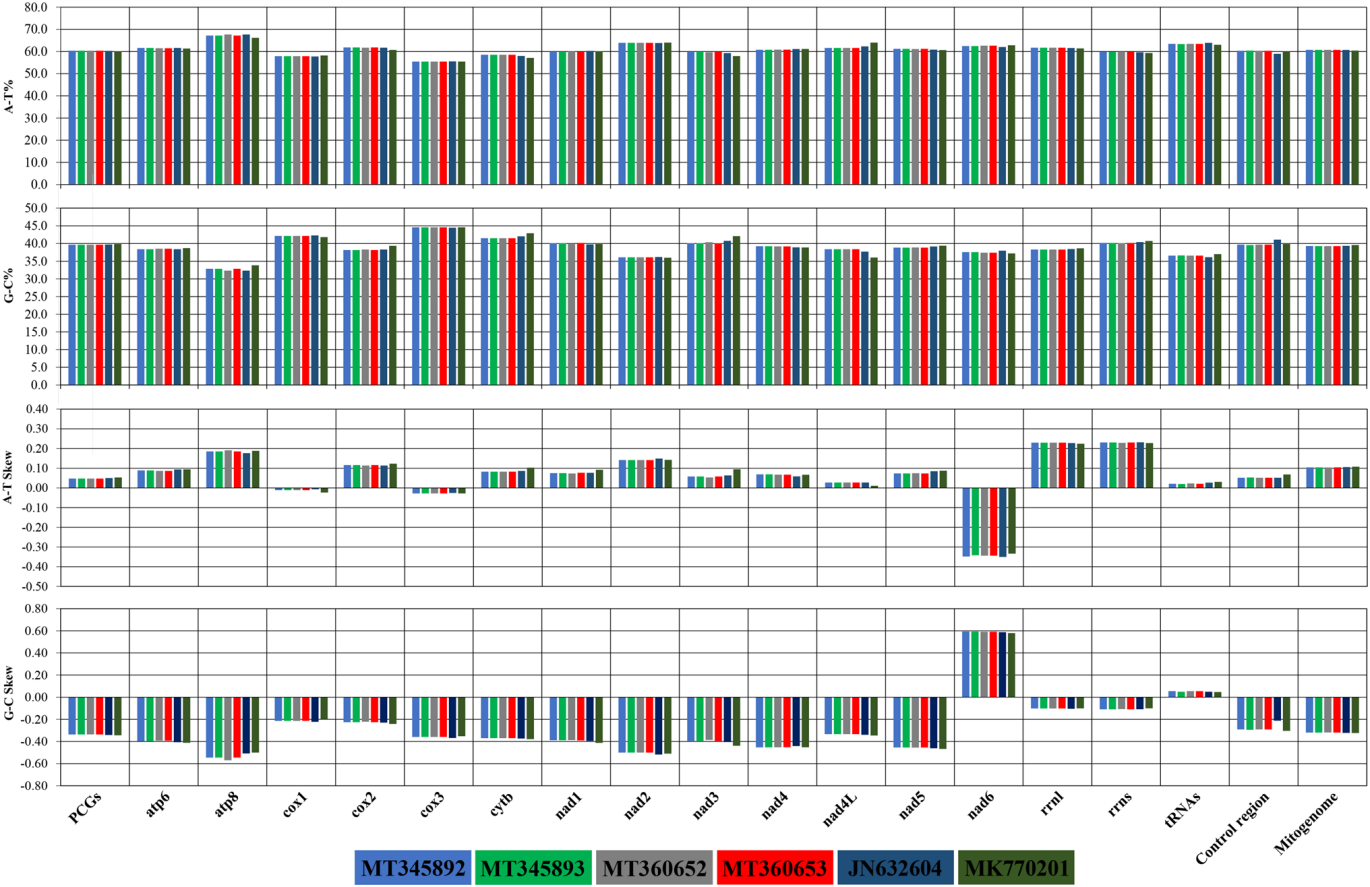
The AT and GC content and skewness of Indian, Cambodian and Malayan gaur mitochondrial genome. MT345892, MT345893, MT360652, MT360653-Indian gaur; JN632604-Cambodian gaur; MK770201-Malayan gaur. The figure was edited in Inkscape 1.0. (https://inkscape.org).

We have sequenced the complete mitochondrial genome of four Indian gaur using two different approaches. The complete mitochondrial genome of Indian gaur was 16,345 bp in length, which included 22 tRNA genes, 13 protein coding genes, 2 ribosomal RNA genes, and a control region (Table 1 and Fig. 1). The genome size and gene order were similar to previously reported gaur and mithun mitochondrial genomes^17,18^. The heavy (H) strand encoded most of the genes (28 of the 37 genes) except NADH dehydrogenase subunit 6 (*nad6*) and 8 tRNA genes (*trnQ, trnA, trnN, trnC, trnY, trnS2, trnE* and *trnP)* which were encoded by the Light (L) strand. The AT and GC content of the mitochondrial genome was observed to be 60.7% and 39.3% respectively, which indicated that the nucleotide composition is overall biased towards adenine and thymine (Fig. 2). This is a common trend among bovine species including mithun^16,18,31,32^. Moreover, the mitochondrial genome showed positive AT (0.104), and negative GC (-0.319) skews (Fig. 2) which suggested the higher content of adenine and cytosine than their respective complementary nucleotides guanine and thymine. In total there were 72 overlapping nucleotides in the range from 1 to 40 bp, which were found at 7 distinct locations. The largest overlapping region (40 bp) was observed between the two protein coding genes ATP synthase F0 subunit 8 (*atp8*) and ATP synthase F0 subunit 6 (*atp6*). In addition to these, the intergenic spacer (IGS) was interspersed at 14 regions across the mitochondrial genome with varying range from 1 to 32 bp which summed up to a total length of 75 bp. The largest intergenic spacer was located between the two tRNA genes *trnN* and *trnC*.

**Table 1:** Characteristic features of mitochondrial genome of Indian gaur.

Genes	Strand	Position	Size (bp)	Intergenic spacer (bp)	Anticodon
*trnF*	H	1–67	67	0	GAA/AAA
*rrnS*	H	68–1,023	956	0	
*trnV*	H	1,024–1,090	67	0	TAC
*rrnL*	H	1,091–2,659	1569	0	
*trnL2*	H	2,660–2,734	75	2	TAA
*nad1*	H	2,737–3,692	956	0	
*trnI*	H	3,693–3,761	69	− 3	GAT
*trnQ*	L	3,759–3,830	72	2	TTG
*trnM*	H	3,833–3,901	69	0	CAT
*nad2*	H	3,902–4,943	1,042	0	
*trnW*	H	4,944–5,010	67	1	TCA
*trnA*	L	5,012–5,080	69	1	TGC
*trnN*	L	5,082–5,154	73	32	GTT
*trnC*	L	5,187–5,253	67	0	GCA
*trnY*	L	5,254–5,321	68	1	GTA
*cox1*	H	5,323–6,867	1545	− 3	
*trnS2*	L	6,865–6,935	71	5	TGA
*trnD*	H	6,941–7,008	68	1	GTC
*cox2*	H	7,010–7,693	684	3	
*trnK*	H	7,697–7,763	67	1	TTT
*atp8*	H	7,765–7,965	201	− 40	
*atp6*	H	7,926–8,606	681	− 1	
*cox3*	H	8,606–9,389	784	0	
*trnG*	H	9,390–9,458	69	0	TCC
*nad3*	H	9,459–9,805	347	0	
*trnR*	H	9,806–9,874	69	0	TCG
*nad4l*	H	9,875–10,171	297	− 7	
*nad4*	H	10,165–11,542	1,378	0	
*trnH*	H	11,543–11,612	70	0	GTG
*trnS1*	H	11,613–11,672	60	1	GCT
*trnL1*	H	11,674–11,743	70	0	TAG
*nad5*	H	11,744–13,564	1821	− 17	
*nad6*	L	13,548–14,075	528	0	
*trnE*	L	14,076–14,144	69	4	TTC
*Cob*	H	14,149–15,288	1,140	4	
*trnT*	H	15,293–15,361	69	− 1	TGT
*trnP*	L	15,361–15,426	66	17	TGG
Control region		15,444–16,345	902		

Of the four gaur samples three had anticodon GAA for *trnF* while the sample GR01 had anticodon AAA for *trnF*. The (+) and (−) values in intergenic spacer column represent intergenic nucleotides and overlapping regions between the genes respectively.

### Protein coding genes (PCGs)

**Figure 3:**
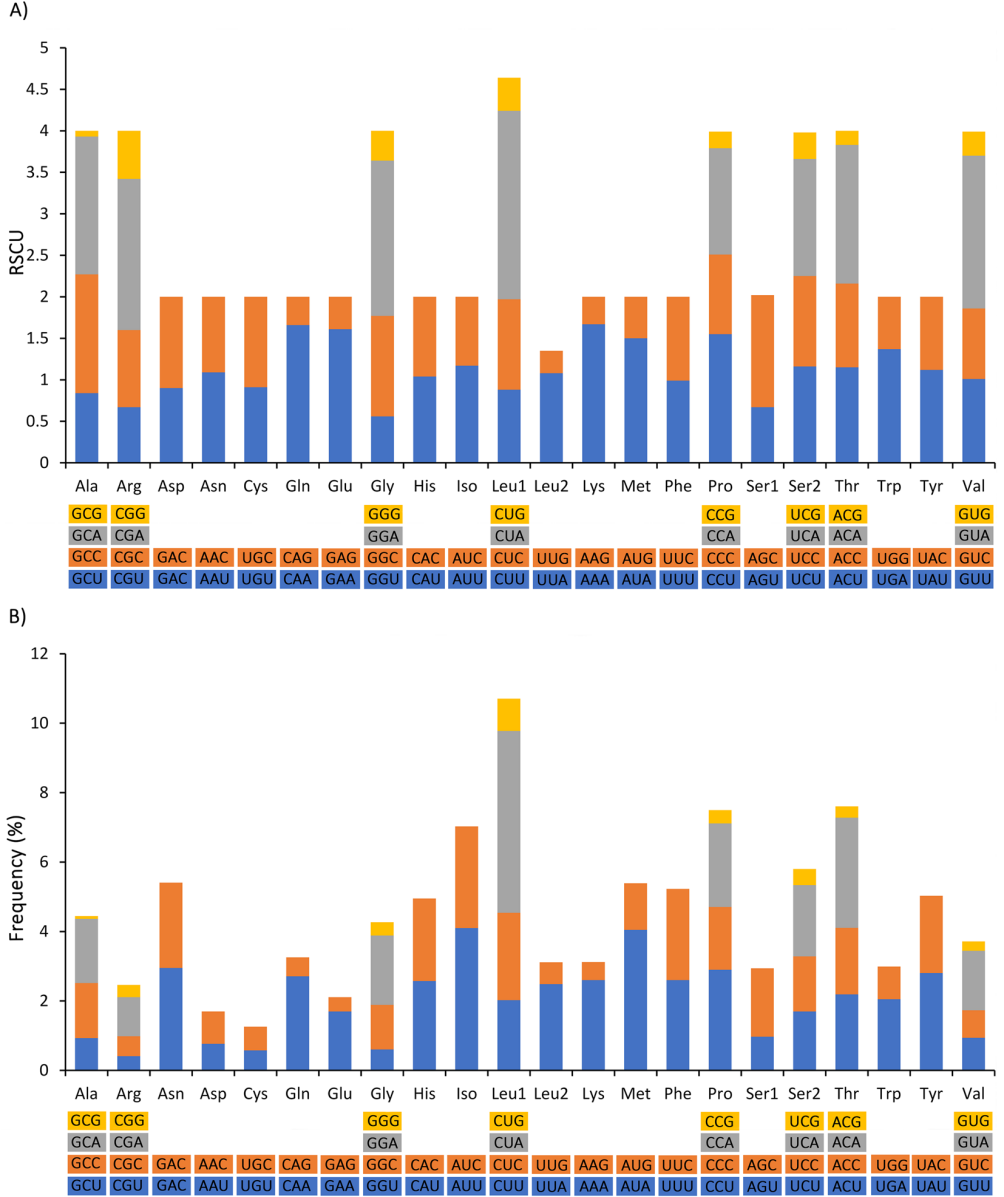
Codon usage of the mitochondrial protein coding genes of Indian gaur (**A**). Relative synonymous codon usage (**B**). Codon usage frequency. Codon families are plotted on the X axis and represented by different color. The RSCU and codon usage frequency are plotted on the Y axis of figure (**A**) and (**B**) respectively. The figure was edited in Inkscape 1.0. (https://inkscape.org).

Mitochondrial genome encoded 13 PCGs and was observed to be 11,339 bp in length which accounted for 69.37% of the mitochondrial genome. The AT and GC content was 60.4% and 39.6% respectively (Fig. 2; Supplementary Information Table S2) which revealed the nucleotide compositional biasness of the PCGs towards adenine and thymine. Moreover, the AT and GC skews of PCGs were positive (0.047) and negative (-0.336) respectively as observed in the case of whole mitochondrial genome (Fig. 2) which suggested that adenine content is relatively higher than thymine while cytosine content is higher than guanine. Also, notably, all the PCGs showed positive AT skew except for the genes *cox1, cox3*, and *nad6*, whereas GC skew was positive only in *nad6* gene. As commonly observed in other bovine and vertebrate species^18,19,32,33^, the PCGs were subdivided into seven NADH dehydrogenase subunits (*nad1*, *nad2*, *nad3*, *nad4*, *nad4L*, *nad5* and *nad6*), three cytochrome *c* oxidase (*cox1*, *cox2* and *cox3*), two ATPase subunits (*atp8* and *atp6*) and a cytochrome *b* gene (*cob*). The size of the PCGs varied significantly with *atp8* (201 bp) being the shortest and *nad5* (1821 bp) being the longest among all. Besides, there were four adjacent pairs of PCGs (*atp8*-*atp6, atp6-cox3, nad4L-nad4* and *nad5-nad6)* which is a common gene positioning observed among the vertebrates^18,19,33^. The relative synonymous codon usage (RSCU) analysis showed the highest utilization of CUA, GGA, GUA and CGA codons among PCGs (Fig. 3A). From the RSCU pattern, it could be understood that among the synonymous alternative codons of each amino acid, the codons associated with adenine at their third codon position were more preferred. The amino acids leucine, threonine, proline and isoleucine were the most abundant among the PCGs (Fig. 3B).

### Ribosomal RNA and transfer RNA

**Figure 4:**
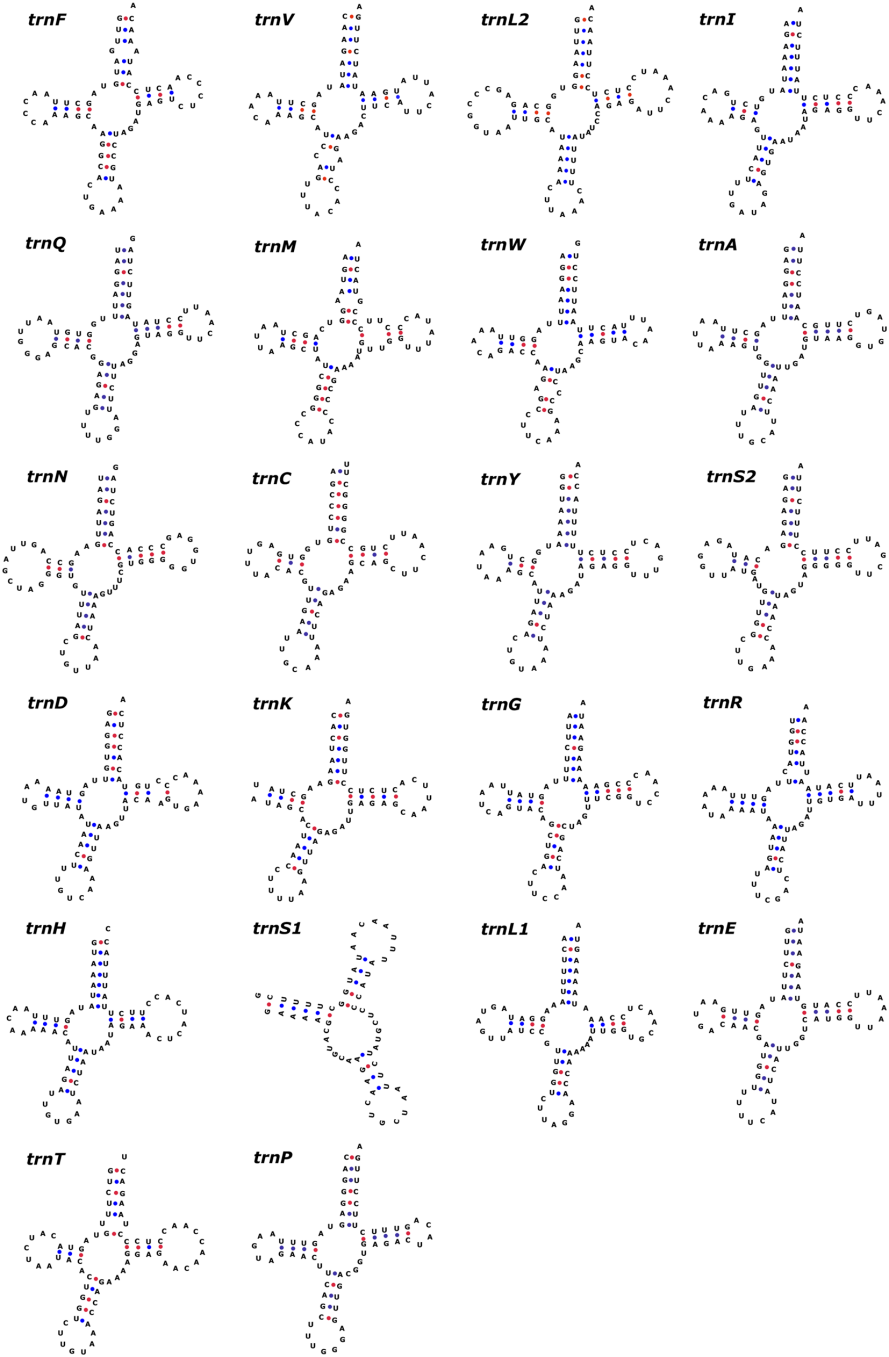
The predicted secondary structures of 22 typical tRNA genes of Indian gaur. Base pairing between G and C is represented by red dots whereas base pairing between A and U is represented by blue dots. Of the four gaur samples three had anticodon GAA for *trnF* while the sample GR01 had anticodon AAA for *trnF* and no other structural difference was observed between the samples. The tRNA structures were generated using the web server tRNAscan-SE^26^ and edited in Inkscape 1.0. (https://inkscape.org).

The total size of the rRNA was 2,525 bp which was formed of two subunits 12S rRNA (956 bp) and 16S rRNA (1569 bp). The nucleotide composition of 12S rRNA as well as 16S rRNA exhibited biasness towards AT content. The AT skew of 12S rRNA and 16S rRNA was positive whereas GC skew was negative, which showed the relatively higher occurrence of adenine and cytosine than thymine and guanine in the rRNAs (Fig. 2). There were 22 tRNA genes in the mitochondrial genome which varied in size from 60 bp (*trnS1*) to 75 bp (*trnL2*). Most of the tRNA genes were encoded by the H-strand (*trnF*, *trnV*, *trnL2*, *trnI*, *trnM*, *trnW*, *trnD*, *trnK*, *trnG*, *trnR*, *trnH*, *trnS1*, *trnL1*, *trnT*) while the tRNAs *trnQ*, *trnA*, *trnN*, *trnC*, *trnY*, *trnS2*, *trnE* and *trnP* were encoded by the L-strand. All the tRNA genes exhibited the cloverleaf secondary structure except *trnS1* and *trnK* which lacked a stable dihydrouridine arm loop (Fig. 4). Such unusual tRNA structures have been commonly observed in mammals including the closely related domestic mithun^18,34^. The AT and GC skews were positive for the tRNAs, which revealed higher compositional count of adenine and guanine than their respective complementary nucleotides (Fig. 2).

### Control region

The control region of the mitochondrial genome was 902 bp in length which was positioned between the tRNAs *trnP* and *trnF*. The AT and GC skews were positive and negative respectively (Fig. 2). The palindromic motifs ‘TACAT’ and ‘ATGTA’ that tend to form hairpin loop structures were found dispersed in the control region in multiple copies. Such identical motifs have also been observed in domestic mithun and other closely related bovine species as well as in other vertebrates^18,19,35,36^, and these are believed to function as the termination site for the elongation of heavy strand^37^. The control region terminated with a characteristic poly-C stretch (13 nucleotides) which was also observed in domestic mithun^18^ (Supplementary Information Table S3).

### Phylogenetic analysis

Overall, there were no differences in size, and gene order and organization among the four mitochondrial genome sequences of Indian gaur. But they differed in nucleotide composition, as there were 33 substitutions for the entire 16,345 bp long mitochondrial genome. It shows extremely low levels of genetic diversity among Indian gaur even though the four samples were collected from far distant places. Our result is in line with a recent study which also observed low genetic diversity among gaur populations of Central India based on partial mtDNA D-loop sequences^38^. Similar low mtDNA diversity was also observed in Malayan gaur for partial D-loop sequence^39^. Low mtDNA diversity is likely to occur in the wild population due to the presence of small number of founder females^38–41^. Also, it can be taken as a sign of population decline^40,42,43^. Genetic variation between individuals is prerequisite for evolution and adaptive changes, and has profound implications for conservation^41,44–46^. The global gaur population is anticipated to fall by 30% within next three decades^3^ due to habitat loss, poaching for its meat and horns and fatal diseases. However, the decline of gaur population in India is considerably lower as compared to other countries such as Bangladesh, Cambodia, China, Laos, Malaysia, Vietnam, and Thailand. Yet the low genetic diversity estimates indicate that a substantial effort needs to be taken immediately to design and implement strategies for the conservation of threatened Indian gaur germplasm. Further analyses using SNP and microsatellite markers may reveal exactly the current status and population structure of Indian gaur.

**Figure 5:**
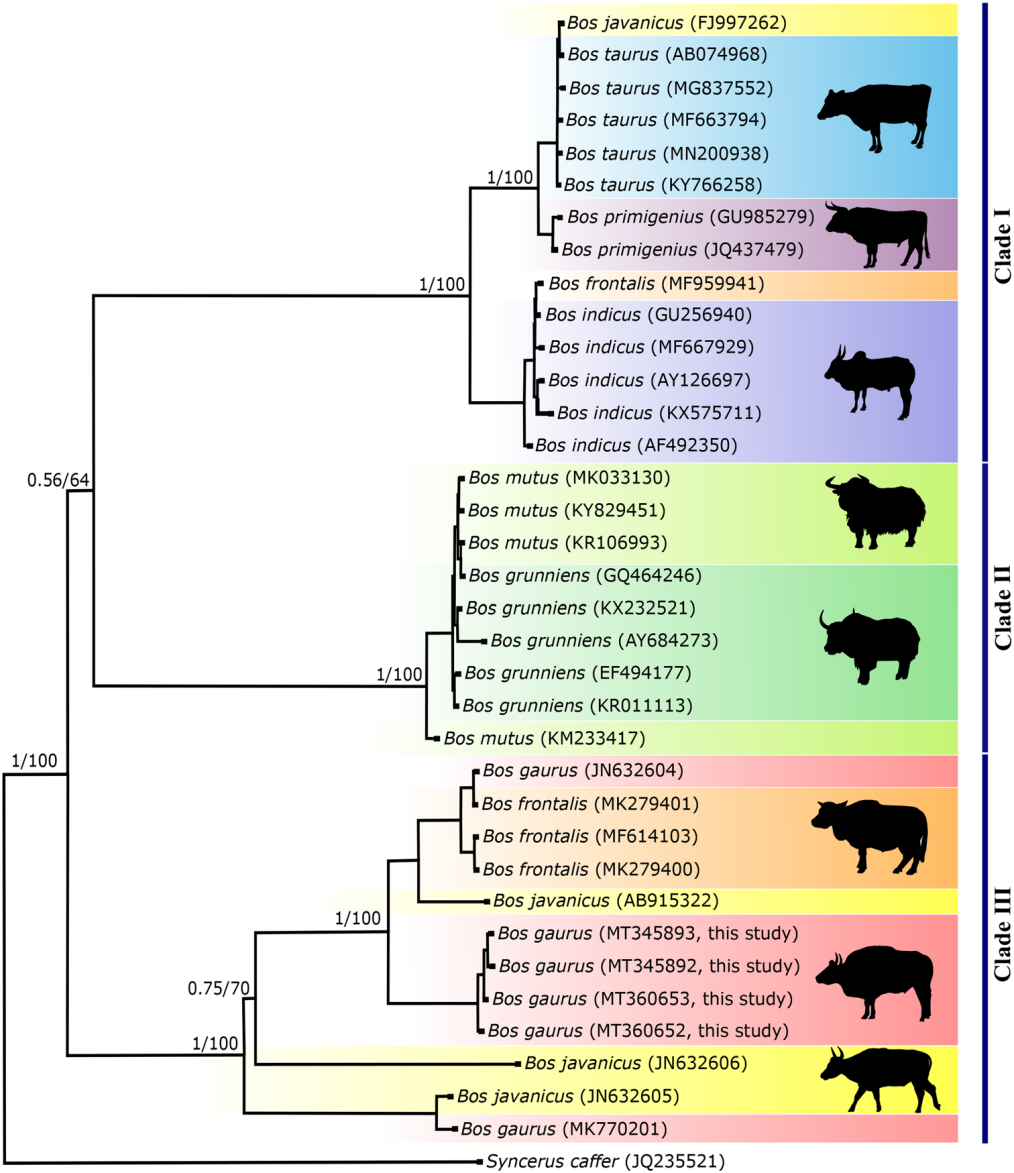
Phylogenetic relationship of *Bos* species inferred from whole mitochondrial genome. Bayesian and maximum parsimony trees were constructed using 16,345 bp sequences of eight *Bos* species. The *Syncerus caffer* mitochondrial genome sequence was used as an out-group. Numbers adjacent to the nodes represent Bayesian posterior probability and bootstrap values. Mitochondrial genome sequences except Indian gaur sequences were retrieved from GenBank and their accession numbers are given in parentheses. The tree was generated using the softwares MEGA^27^and MrBayes^29^. The pictures of different cattle species given in the tree was drawn using Inkscape 1.0. (https://inkscape.org). The figure was edited in Inkscape 1.0.

The Indian gaur mitochondrial genomes were further compared with the mitochondrial genome of Cambodian gaur (JN632604) and Malayan gaur (MK770201). The Cambodian gaur showed 304 substitutions with Indian gaur whereas 634 substitutions were observed between Indian and Malayan gaur. The AMOVA revealed 94% variation between Indian and Cambodian gaur and that between Indian and Malayan gaur was 97%, which indicated that there is significant genetic variation among Indian, Cambodian and Malayan gaur. Thus, in order to understand the phylogenetic relationship of Indian, Cambodian and Malayan gaur, phylogenetic trees were constructed along with seven congeneric species viz* B. frontalis, B. taurus, B. indicus, B. javanicus, B. mutus, B. grunniens* and *B. primigenius*. For the congeneric species, a maximum of five sequences for each species or total available sequences were included in the analysis. The African buffalo, *Syncerus caffer* was used as an out-group. Bayesian phylogenetic tree showed three distinct clades and each clade was further divided by the wild and domestic individual. The MP tree was identical to the former, with strong bootstrap support. The clade one included wild and domestic cattle while the clade two was fully encompassed with wild and domestic yak. Similar to clade one and two, clade three comprised of wild gaur and domestic mithun which demonstrated the ancestral connections between gaur and domestic mithun (Fig. 5). The banteng, *B. javanicus* was distributed in all the clades except clade 2 along with taurine cattle, domestic mithun and gaur which shows the hybrid nature of *B. javanicus* as reported elsewhere^47^. The presence of wild gaur and domestic mithun in a single clade as observed in the case of cattle and yak (Fig. 5) unambiguously suggests that wild gaur is the maternal ancestor of domestic mithun. Similar findings have also been obtained in previous studies using 16S rRNA gene^10^, SNP^11^, cytochrome *b* gene^48,49^ and Y chromosomal DNA^50^ markers. Further, AMOVA revealed less (34%) variation between mithun and gaur as compared to mithun and cattle (> 97%). Genetic divergence was also much lower (0.031) between gaur and mithun than between mithun and the other two cattle species ($\ge$ 0.052). Moreover, studies based on descriptive characteristics, protein polymorphism, karyotype, and microsatellite marker have showed significant differences between domestic mithun and cattle^11,51–55^. Therefore, based on the results of present and previous studies, we strongly suggest that, wild gaur is the maternal ancestor of domestic mithun.

On the other hand, one of the Chinese mithun clustered with *B. indicus* (Fig. 5)*,* but its nuclear genome did not support this clustering^13^. Introgression of cattle mitochondrial DNA into mithun has been reported by Li et al*.*^48^ and Gou et al.^56^. These outcomes demonstrate that some mithun have mithun chromosomal genome and cattle mitochondrial genome. Hybridization has been practiced between mithun and cattle to obtain animal of higher economic value since historical times^51^. Studies have also reported the transportation of mithun from India to Bhutan during the ancient times, where they were extensively used to cross breed with domestic cattle^57^. It is therefore, possible that mithun individuals analysed in these studies were likely to be the hybrid of domestic mithun and domestic cattle which is prevalent in China^16,56^. Thus, in domestic animals phenotype is not always related to their mitochondrial genome^58^, which leads to phenotypic ambiguity among domestic species and impede conservation efforts. Identification of genetically unique population is critical for species conservation hence, an extensive study is essential to evaluate the cross bred animals on a larger scale using both chromosomal and mitochondrial DNA markers.

The Indian gaur, Cambodian gaur and Malayan gaur formed distinct clades within the third clade with strong Bayesian posterior probability and bootstrap values (Fig. 5). Our finding therefore, clearly supports the existence of three sub species of gaur i.e. *B. gaurus gaurus, B. gaurus readei* and *B. gaurus hubbacki* proposed based on the morphological features by Lydekker^4,5^. Further, the phylogenetic tree revealed that among three subspecies, the *B. gaurus gaurus* is genetically closer to *B. gaurus readei* as compared to *B. gaurus hubbacki*. Nevertheless more number of samples from each subspecies needs to be analysed using both the nuclear and mitochondrial DNA markers to confirm their identity. Also, the domestic mithun including Indian mithun samples were clustered with Cambodian gaur which explains the close genetic relationship of domestic mithun and Cambodian gaur. However, at this juncture it is premature to suggest the place of domestication of mithun while considering the sample size of the present study particularly the gaur of Northeast India which is reported to closely resemble the Southeast Asian gaur^7^ would have significant implications on the origin of domestic mithun. On the whole, we suggest that it is not only important but necessary to conduct a detailed study on complete mitochondrial genome of gaur from different countries particularly Northeast India, Myanmar, Malaysia and China to unveil the time and place of domestication of mithun. On the other hand, studies on whole mitochondrial genome of domestic mithun from different countries would further complement to establish the domestication history of mithun.

## Conclusions

The study on complete mitochondrial genome of Indian gaur is of paramount importance primarily for two reasons (i) gaur is considered as the ancestral species of domestic mithun but still is in dispute and (ii) there is a taxonomic ambiguity with regard to the classification of gaur into sub species. This is the first study to sequence the complete mitochondrial genome of Indian gaur. The findings of our study clearly conclude that, gaur is the maternal ancestor of domestic mithun. Although, Indian gaur mitochondrial genome shared similar structural features with Cambodian and Malayan gaur, a significant genetic variation was observed between them which support the existence of three sub species of gaur. Further, the low genetic diversity of Indian gaur indicates the necessity for developing appropriate conservation strategies for gaur populations in India. The complete mitochondrial genome sequence of gaur would serve as a useful genetic resource for phylogenetic, evolutionary biology, and conservation related studies.

## Supplementary information

Supplementary information.

## Data Availability

The assembled mitochondrial genome sequences are available at GenBank under the following Accession Nos. MT345892, MT345893, MT360652 and MT360653. The raw sequence reads can be found at Sequence Read Archive (SRA) under the BioProject ID: PRJNA627336.
