# Supplementary material for: The complete mitochondrial genome of Indian gaur, Bos gaurus and its phylogenetic implications
Source: Sci Rep. 2020 Jul 20;10:11936. doi: 10.1038/s41598-020-68724-6 (PMC7371690; doi:10.1038/s41598-020-68724-6)
Supplement: Supplementary file 1 — Supplementary information. [file 41598_2020_68724_MOESM1_ESM.pdf]

The complete mitochondrial genome of Indian gaur, *Bos gaurus* and its phylogenetic implications

Ranganathan Kamalakkannan[#](https://orcid.org/0000-0001-8378-4629), Karippadakam Bhavana[#](https://orcid.org/0000-0002-8287-9957), Vandana R Prabhu[#] (https://orcid.org/0000-0002-6302-8022), Dhandapani Sureshgopi, Hijam Surachandra Singha (https://orcid.org/0000-0002-3108-0846) and Muniyandi Nagarajan* (https://orcid.org/0000-0001-8328-9767)

Department of Genomic Science

School of Biological Sciences

Central University of Kerala

Kasaragod-671316, Kerala, India

[#]These authors contributed equally to this work

*Corresponding author email: nagarajan@cukerala.ac.in

Table S1. The primers used to amplify the mitogenome for the DNA isolated from the dung samples (Hassanin et al. 2009)[20]

S. No	Primer Sequence	Annealing temperature(°C)
1	1F-ACCATGCCGCGTGAAACCAGCA	54
	1R-GYGYGGATRCTTGCATGTGTA	
2	2F-CACTGAAAATGCCTAGATGAG	55
	2R-CTAGGTGTAAACTAGRTGCTT	
3	3F-GCACGCACACACCGCCCGTCAC	57
	3R-CGCTTTCTTAATTGRTGGCTGC	

4	4F-AGCCTGGTGATAGCTGGTTGTCC	51
	4R-AAGCTCCATAGGGTCTTCTCGTC	
5	5F-CCGTGCAAAGGTAGCATAATCA	55
	5R-CCTAGNACTTTTCGTTCNACTA	
6	6F-GTGGCAGAGCCCGGTAATTG	51
	6R-TTACTCTATCAAAGTAACTC	
7	7F-TYCGAGCATCHTAYCCHCGATT	55
	7R-TGGTTTAGBCCBCCTCAKCCYCC	
8	8F-CACTTYTGAGTNCCAGAAGT	56
	8R-TAGGGTRTTTAGCTGTTAAC	
9	9F-AGACCAAGAGCCTTCAAAGC	55
	9R-GCTTCWACTATDGADGATGC	
10	10F-GGNGGNTTYGGHAAYTGACT	55
	10R-GAARATRAAGCCTAGRGCTCA	
11	11F-TTYACHGTHGGAATAGAYGT	46°C
	11R-GCRTCTTGRAANCCTARTTG	
12	12F-CCCCCYAYWRYTGGTTTCAAGCCA	55°C
	12R-GTKGAYGTRTCTAGTTGYGGCAT	
13	13F-CAATGCTCHGARATYTGYGG	56.5
	13R-GANARDGCTCCYGTDAGNGGTCA	
14	14F-GCCTAYGTNTTYACYCTNCTAGT	55
	14R-TGATTGGAAGTCARYTGTAC	
15	15F-GTHTCYATCTATTGATGAGG	48
	15R-CAGGTYAGRGGDATDAGTAT	
16	16F-AGCYTGYGAAGCAGCACTAGG	55
	16R-GCKGTRGCTCCTATRTARCTTCA	
17	17F-AGCTCHATYTGYYTHCGYCAAAC	55°C
	17R-CCAATTTTTTGGYTCCTAAGRCC	
18	18F-CCGAAAAAGYAYGCAAGAACTGC	55°C
	18R-GCDGATTTTCCDGTKGCDGCTA	
19	19F-GACGARCAGAYGCHAAYACAGC	55
	19R-GTDAKTADDAGGGCTCAGGCG	
20	20F-GGMAGCCTNGCNYTAACAGG	46
	20R-AGTTTAATGGDHTDGGDGATTG	
21	21F-CCATAACTRTAYAAAGCHGCAA	55°C
	21R-CCTCARAATGATATTTGKCCTCA	
22	22F-CAGGMCTATTCCTRGCHATACA	59
	22R-CCCTTYTCTGGTTTACAAGACC	
23	23F-CATCGGACAACTAGCATCTAT	53
	23R-CCTGAAGWAAGAACCAGATG	

Table S2. The AT and GC content and skewness of Indian, Cambodian and Malayan gaur mitochondrial genome

AT content

GenBank Accession No.	PCGs	atp6	atp8	cox1	cox2	cox3	cytb	nad1	nad2	nad3
MT345892	60.36	61.62	67.16	57.86	61.84	55.43	58.51	59.94	63.92	59.94
MT345893	60.36	61.62	67.16	57.86	61.84	55.43	58.51	59.94	63.92	59.94
MT360652	60.36	61.47	67.66	57.86	61.70	55.43	58.51	60.04	63.92	59.65
MT360653	60.36	61.47	67.16	57.86	61.84	55.43	58.51	59.83	63.92	59.94
JN632604	60.28	61.62	67.66	57.73	61.70	55.56	57.98	60.25	63.82	59.25
MK770201	60.16	61.31	66.17	58.19	60.67	55.43	57.11	60.15	64.01	57.93

GenBank Accession No.	nad4	nad4L	nad5	nad6	rrnl	rrns	tRNAs	Control region	Mitogenome
MT345892	60.76	61.62	61.18	62.43	61.70	59.83	63.43	60.31	60.73
MT345893	60.76	61.62	61.18	62.43	61.70	59.83	63.36	60.42	60.74
MT360652	60.83	61.62	61.12	62.62	61.70	59.94	63.43	60.31	60.74
MT360653	60.83	61.62	61.18	62.62	61.70	59.83	63.43	60.31	60.73
JN632604	61.09	62.29	60.85	62.04	61.57	59.62	63.88	58.92	60.67
MK770201	61.12	63.97	60.63	62.82	61.36	59.31	63.01	60.11	60.43

GC- content

GenBank Accession No.	PCGs	atp6	atp8	cox1	cox2	cox3	cytb	nad1	nad2	nad3
MT345892	39.64	38.38	32.84	42.14	38.16	44.57	41.49	40.06	36.08	40.06
MT345893	39.64	38.38	32.84	42.14	38.16	44.57	41.49	40.06	36.08	40.06
MT360652	39.64	38.53	32.34	42.14	38.30	44.57	41.49	39.96	36.08	40.35
MT360653	39.64	38.53	32.84	42.14	38.16	44.57	41.49	40.17	36.08	40.06
JN632604	39.72	38.38	32.34	42.27	38.30	44.44	42.02	39.75	36.18	40.75
MK770201	39.84	38.69	33.83	41.81	39.33	44.57	42.89	39.85	35.99	42.07

GenBank Accession No.	nad4	nad4L	nad5	nad6	rrnl	rrns	tRNAs	Control region	Mitogenome
MT345892	39.24	38.38	38.82	37.57	38.30	40.17	36.57	39.69	39.27
MT345893	39.24	38.38	38.82	37.57	38.30	40.17	36.64	39.58	39.26
MT360652	39.17	38.38	38.88	37.38	38.30	40.06	36.57	39.69	39.26
MT360653	39.17	38.38	38.82	37.38	38.30	40.17	36.57	39.69	39.27
JN632604	38.91	37.71	39.15	37.96	38.43	40.38	36.12	41.08	39.33
MK770201	38.88	36.03	39.37	37.18	38.64	40.69	36.99	39.89	39.57

AT -skewness

GenBank Accession No.	PCGs	atp6	atp8	cox1	cox2	cox3	cytb	nad1	nad2	nad3
MT345892	0.04734	0.08861	0.18519	-0.01119	0.11584	-0.02765	0.08246	0.07504	0.14114	0.05769
MT345893	0.04763	0.08861	0.18519	-0.01119	0.11584	-0.02765	0.08246	0.07504	0.14114	0.05769
MT360652	0.04705	0.08629	0.19118	-0.01119	0.11374	-0.02765	0.08246	0.07317	0.14114	0.05314
MT360653	0.04734	0.08629	0.18519	-0.01119	0.11584	-0.02765	0.08246	0.07692	0.14114	0.05769
JN632604	0.05004	0.09367	0.17647	-0.00673	0.11374	-0.02529	0.08623	0.07639	0.14887	0.06341
MK770201	0.05292	0.09415	0.18797	-0.02336	0.12289	-0.02765	0.10292	0.09217	0.14243	0.09453

GenBank Accession No.	nad4	nad4L	nad5	nad6	rrnl	rrns	tRNAs	Control region	Mitogenome
MT345892	0.06843	0.02732	0.07361	-0.34796	0.22934	0.23077	0.02096	0.05147	0.10417
MT345893	0.06843	0.02732	0.07361	-0.34169	0.22934	0.23077	0.01994	0.05321	0.10415
MT360652	0.06715	0.02732	0.07457	-0.34375	0.22934	0.22862	0.02306	0.05147	0.10395
MT360653	0.06715	0.02732	0.07361	-0.34375	0.22934	0.23077	0.02096	0.05147	0.10406
JN632604	0.05854	0.02703	0.08484	-0.35016	0.22774	0.23158	0.02697	0.05164	0.10578
MK770201	0.06683	0.01053	0.08696	-0.33333	0.22407	0.22751	0.03062	0.06839	0.10747

GC-skewness

GenBank Accession No.	PCGs	atp6	atp8	cox1	cox2	cox3	cytb	nad1	nad2	nad3
MT345892	-0.33660	-0.39837	-0.54545	-0.21352	-0.22605	-0.35817	-0.36998	-0.38903	-0.50000	-0.39568
MT345893	-0.33660	-0.39837	-0.54545	-0.21352	-0.22605	-0.35817	-0.36998	-0.38903	-0.50000	-0.39568
MT360652	-0.33615	-0.39271	-0.56923	-0.21352	-0.22137	-0.35817	-0.36998	-0.38743	-0.50000	-0.38571
MT360653	-0.33660	-0.39271	-0.54545	-0.21352	-0.22605	-0.35817	-0.36998	-0.39063	-0.50000	-0.39568
JN632604	-0.34088	-0.40650	-0.50769	-0.22205	-0.22901	-0.36782	-0.37370	-0.39474	-0.51724	-0.40426
MK770201	-0.34351	-0.41129	-0.50000	-0.19814	-0.24164	-0.35244	-0.37832	-0.41207	-0.50933	-0.43836

GenBank Accession No.	nad4	nad4L	nad5	nad6	rrnl	rrns	tRNAs	Control region	Mitogenome
MT345892	-0.45353	-0.33333	-0.45403	0.59375	-0.10150	-0.10938	0.05455	-0.29050	-0.31983
MT345893	-0.45353	-0.33333	-0.45403	0.59375	-0.10150	-0.10938	0.04900	-0.29412	-0.31962
MT360652	-0.45251	-0.33333	-0.45480	0.59162	-0.10150	-0.10705	0.05455	-0.29050	-0.31868
MT360653	-0.45251	-0.33333	-0.45403	0.59162	-0.10150	-0.10938	0.05455	-0.29050	-0.31910
JN632604	-0.44090	-0.33929	-0.46143	0.58763	-0.10448	-0.10881	0.04954	-0.21212	-0.32203
MK770201	-0.45216	-0.34579	-0.46722	0.57895	-0.10049	-0.10026	0.04676	-0.30362	-0.32335

Table S3. Control region sequence of Indian gaur

> MT345892
AACACTATTAATATAGTTCCACAAACGCAAAGAGCTTTACCAGTATTAAATTTAT
CAAAAATTTCAACAACTCAACACAGACTTTATACTCTAACCAAATATCACAAACA
CCACTAGCTAATAACACACGCCCTCCCGCACACTACAAAATATACTCTTCACAGC
AGGATACGTACATAATATTAATGTAATAAAGACATAATATGTATATAGTACATTA
TATTATATGCCCCATGCATATAAGCAAGTACTTGAACTTCATAGACAGTACATAG
TACATGAACTCATTAATCGTACATAGCACATTATGTCAAATTCATCCTTGACAGC
ATGCGTATCCCTGCCATTAGATCACGAGCTTAATTACCATGCCGCGTGAAACCAG
CAACCCGCTAGGCAGAGGATCCCTCTTCTCGCTCCGGGCCCATAAACTGTGGGGG
TCGCTATTTAATGAACTTTATCAGACATCTGGTTCTTTCTTCAGGGCCATCTCATC
TAAAACCGTCCACTCTTTCCTCTTAAATAAGACATCTCGATGGACTAATGACTAA
TCAGCCCATGCTCACACATAACTGTGCTGTCATACATTTGGTATTTTTTTATTTTG
GGGGATGCTTGGACTCAGCTATGGCCGTCAGAGGCCCTGACCCGGAGCATTTATT
GTAGCTGGACTTAACTGCATCTTGAGCACCAGCATAATGATAGGCATGGACATTA
CAGTCAATGGTCACAGGACATAAATTATATTATATATCCCCCCTTCCATAAAACT
TCCCCCTTAAATATTTACCACCACTTTTAACAGACTTTCCCCTAGTTACTTGTTTG
AATTTTCCACGATTTCAATACTCAAATTAGCACTCTAAATAAAGTCAATATATAA
ACGCGGCCCCCCCCCCCC

> MT345893
AACACTATTAATATAGTTCCACAAACGCAAAGAGCTTTACCAGTATTAAATTTAT
CAAAAATTTCAACAACTCAACACAGACTTTATACTCTAACCAAATATCACAAACA
CCACTAGCTAATAACACACGCCCTCCCGCACACTACAAAATATACTCTTCACAGC
AGGATACGTACATAATATTAATGTAATAAAGACATAATATGTATATAGTACATTA
TATTATATGCCCCATGCATATAAGCAAGTACTTGAACTTCATAGACAGTACATAG
TACATGAACTCATTAATCGTACATAGCACATTATGTCAAATTCATCCTTGACAGC
ATGCGTATCCCTGCCATTAGATCACGAGCTTAATTACCATGCCGCGTGAAACCAG
CAACCCGCTAGGCAGAGGATCCCTCTTCTCGCTCCGGGCCCATAAACTGTGGGGG
TCGCTATTTAATGAACTTTATCAGACATCTGGTTCTTTCTTCAGGGCCATCTCATC
TAAAACCGTCCACTCTTTCCTCTTAAATAAGACATCTCGATGGACTAATGACTAA
TCAGCCCATGCTCACACATAACTGTGCTGTCATACATTTGGTATTTTTTTATTTTG
GGGGATGCTTGGACTCAGCTATGGCCGTCAGAGGCCCTGACCCGGAGCATTTATT
GTAGCTGGACTTAACTGCATCTTGAGCACCAGCATAATGATAGGCATGGACATTA

CAGTCAATGGTCACAGGACATAAATTATATTATATATCCCCCCTTCCATAAAACT
TCCCCCTTAAATATTTACCACCACTTTTAACAGACTTTCCCCTAGTTACTTGTTTG
AATTTTCCACGATTTCAATACTCAAATTAGCACTCTAAATAAAGTCAATATATAA
GCGCGGCCCCCCCCCCCCC

> MT360652
AACACTATTAATATAGTTCCACAAACGCAAAGAGCTTTACCAGTATTAAATTTAT
CAAAAATTTCAACAACTCAACACAGACTTTATACTCTAACCAAATATCACAAACA
CCACTAGCTAATAACACACGCCCTCCCGCACACTACAAAATATACTCTTCACAGC
AGGATATGTACATAATATTAATGTAATAAAGACATAATATGTATATAGTACATTA
TATTATATGCCCCATGCGTATAAGCAAGTACTTGAACTTCATAGACAGTACATAG
TACATGAACTCATTAATCGTACATAGCACATTATGTCAAATTCATCCTTGACAGC
ATGCGTATCCCTGCCATTAGATCACGAGCTTAACTACCATGCCGCGTGAAACCAG
CAACCCGCTAGGCAGAGGATCCCTCTTCTCGCTCCGGGCCCATAAACTGTGGGGG
TCGCTATTTAATGAACTTTATCAGACATCTGGTTCTTTCTTCAGGGCCATCTCATC
TAAAACCGTCCACTCTTTCCTCTTAAATAAGACATCTCGATGGACTAATGACTAA
TCAGCCCATGCCCACACATAACTGTGCTGTCATACATTTGGTATTTTTTTATTTTG
GGGGATGCTTGGACTCAGCTATGGCCGTCAAAGGCCCTGACCCGGAGCATTTATT
GTAGCTGGACTTAACTGCATCTTGAGCACCAGCATAATGATAGGCATGGGCATTA
CAGTCAATGGTTACAGGACATAAATTATATTATATATCCCCCCTTCCATAAAACT
TCCCCCTTAAATATTTACCACCACTTTTAACAGACTTTCCCCTAGTTACTTGTTTA
AATTTTCCACGATTTCAATACTCAAATTAGCACTCTAAATAAAGTCAATATATAA
GCGCGGCCCCCCCCCCCCC

> MT360653
AACACTATTAATATAGTTCCACAAACGCAAAGAGCTTTACCAGTATTAAATTTAT
CAAAAATTTCAACAACTCAACACAGACTTTATACTCTAACCAAATATCACAAACA
CCACTAGCTAATAACACACGCCCTCCCGCACACTACAAAATATACTCTTCACAGC
AGGATACGTACATAATATTAATGTAATAAAGACATAATATGTATATAGTACATTA
TATTATATGCCCCATGCATATAAGCAAGTACTTGAACTTCATAGACAGTACATAG
TACATGAACTCATTAATCGTACATAGCACATTATGTCAAATTCATCCTTGACAGC
ATGCGTATCCCTGCCATTAGATCACGAGCTTAATTACCATGCCGCGTGAAACCAG
CAACCCGCTAGGCAGAGGATCCCTCTTCTCGCTCCGGGCCCATAAACTGTGGGGG
TCGCTATTTAATGAACTTTATCAGACATCTGGTTCTTTCTTCAGGGCCATCTCATC
TAAAACCGTCCACTCTTTCCTCTTAAATAAGACATCTCGATGGACTAATGACTAA
TCAGCCCATGCTCACACATAACTGTGCTGTCATACATTTGGTATTTTTTTATTTTG
GGGGATGCTTGGACTCAGCTATGGCCGTCAGAGGCCCTGACCCGGAGCATTTATT
GTAGCTGGACTTAACTGCATCTTGAGCACCAGCATAATGATAGGCATGGACATTA
CAGTCAATGGTCACAGGACATAAATTATATTATATATCCCCCCTTCCATAAAACT
TCCCCCTTAAATATTTACCACCACTTTTAACAGACTTTCCCCTAGTTACTTGTTTG
AATTTTCCACGATTTCAATACTCAAATTAGCACTCTAAATAAAGTCAATATATAA
GCGCGGCCCCCCCCCCCCC
